# Use of social media for the improvement of safety knowledge and awareness among Saudi Arabian phlebotomists

**DOI:** 10.3389/fmed.2023.1194969

**Published:** 2023-08-03

**Authors:** Razaz Attar, Asmaa Almohanna, Ahlam Almusharraf, Amal Alhazmi, Nouf Alanzi, Fahad Al-Anezi, Turki Alanzi, Raghad Sroor, Ahmed Albishri, Amwaj Alzahrani, Taif Alsabilah, Ali Alkenani, Raghad Alghamdi, Fai AlGethami, Arub AlGethami

**Affiliations:** ^1^Princess Nourah bint Abdulrahman University, Riyadh, Saudi Arabia; ^2^Jouf University, Sakakah, Al Jawf, Saudi Arabia; ^3^Imam Abdulrahman Bin Faisal University, Dammam, Saudi Arabia; ^4^Taibah University, Al Madinah Al Munawwarah, Saudi Arabia; ^5^King Abdulaziz Medical City, Riyadh, Saudi Arabia; ^6^Umm al-Qura University, Mecca, Saudi Arabia; ^7^University of Tabuk, Tabuk, Saudi Arabia; ^8^Obied Specialized Hospital, Riyadh, Saudi Arabia; ^9^Al Baha University, Al Bahah, Saudi Arabia; ^10^Taif University, Ta'if, Saudi Arabia

**Keywords:** social media, phlebotomists, knowledge, awareness, information, sharing

## Abstract

**Purpose:**

The purpose of this study is to investigate the use of social media for the improvement of safety knowledge and awareness among phlebotomists.

**Methods:**

As this study was intended to arrive at specific conclusions using empirical evidence, a deductive quantitative cross-sectional online survey design was adopted. A total of 521 phlebotomists participated in the survey, and 86 incomplete responders were removed, resulting in a final sample of 435 considered in this study. *T*-tests and ANOVA were used to analyze the data.

**Results:**

A total of 41.6% stated that social media was very effective, and 31.5% stated that it was somewhat effective in improving safety knowledge and awareness. in addition, this study revealed no major differences between male and female participants (*p* > 0.05) with respect to the effectiveness of social media. However, statistically significant differences (*p* < 0.05) among the age groups were identified in relation to the effectiveness of social media and the intention to use it in the future.

**Conclusion:**

Social media applications are effective for knowledge dissemination among healthcare professionals.

## Introduction

With the increase in access to the Internet, the number of social media users has been rapidly increasing across the world. According to a study conducted by Kepios (1), 4.76 billion people across the globe are using social media, which represents 59.4% of the global population. According to a recent study (2), the number of social media users globally has increased from 1,720 million in January 2013 to 4,760 million in January 2023, reflecting a 177% increase in a decade. Currently, for every 10 users on the Internet, 9 users are using social media (3). As social media platforms allow only those who are aged above 13 years, it can be understood that 78% of the eligible global population uses social media, indicating high adoption rates of social media among people. Moreover, on average, more than 2.5 h per day is spent on social media by users, reflecting 15% of the waking time spent on social media platforms by the users (1). With 2.9 billion active users, Facebook is the top social media platform, followed by YouTube (2.5 billion), WhatsApp (2 billion), Instagram (2 billion), and Twitter (556 million). It is also identified that most of the social media users use four to seven different social media applications (4). According to GWI (5) report, WhatsApp is the most popular social media platform globally, followed by Instagram, Facebook, WeChat, and TikTok. According to a study conducted by GWI in 2022, the main reasons for using social media included keeping in touch with family and friends, filling spare time, reading new stories, finding content (such as articles and videos), and seeing what is being talked about.

Social media apps have swiftly grown in the professional field as a result of social media's popularity in private usage contexts. These applications are helpful as a gauge for strategic information management in an organization's internal environment. Social media in this regard promises to provide sufficient tools to assist both methodical knowledge storage and sharing of knowledge and communication in businesses. However, because social media is only effective when used, employees' motivation to use it is a crucial factor for increasing knowledge and awareness and supporting their professional growth (6). Several studies (7) on the use of social media in the workplace for work-related use could enhance the employees' knowledge exchange process and also their performance. A study on small- and medium-scale enterprises (SMEs) in Germany (8) has found that internal social media, such as blogs and wikis, are used by companies to enhance collaboration among employees and to improve knowledge management. Similarly, another study in China (9) identified that both work-oriented social media and socialization-oriented social media applications can generate synergies to improve employee performance. Not only performance but also social media was identified to be having positive effects on improving work engagement, satisfaction, and organizational commitment (10). A few studies (11–14) have also highlighted the negative effects of using social media in the workplace. For instance, the issue of privacy and security was highlighted in (11) for influencing information-sharing decisions within organizations. In addition, its effects, such as getting distracted and addicted to the use, can negatively affect employee performance and strain organizational resources (12). Considering the similar effects, a study in Hungary (13) suggested that companies should train their employees and motivate them to use social media for knowledge sharing at workplaces. The need for social media in the workplace is realized in many organizational sectors such as news and media (11), insurance (14), and healthcare (15). A recent study of social media usage by doctors (15) in healthcare organizations identified three themes including, extended communication and relationships among doctors, beneficial for acquiring existing and new knowledge, and knowledge sharing and transfer for promoting knowledge exchange.

The healthcare system in Saudi Arabia is complex compared to other countries as the nation largely depends on expatriate healthcare workers in different streams of healthcare (16). Furthermore, it is observed that the percentage of demand supply of personnel in allied health services is projected to increase from 82.4% in 2020 to 99% in 2030 (17), indicating a severe shortage of allied health workers such as phlebotomists. Furthermore, Saudi Arabia is facing issues in healthcare management with frequent epidemics, such as Ebola, MERS, SARS, and the recent COVID-19 pandemic (18–20), creating a huge burden on the healthcare system. Moreover, the prevalence of chronic diseases, such as diabetes, cancer, and hypertension, is increasing at a high rate in the country. In this context, phlebotomists play an important role in managing public health as they are critical resources in disease diagnosis and management. In addition, issues are observed in Saudi Arabia with phlebotomist staffing ratios, readiness, compliance with guidelines, and awareness (21, 22). Therefore, it is important to develop or utilize technology interventions in increasing the safety knowledge and awareness of phlebotomists in Saudi Arabia.

This widespread social media usage has impacted how information is shared, how people communicate, how knowledge is disseminated, and how users—like patients and doctors—interact with one another (23). The usage of social media has also helped healthcare workers perform better by enhancing their knowledge, skills, competencies, and learning environments (23–25). These tools are also helpful in increasing awareness and assisting with the physical and psychological care of patients suffering from a variety of illnesses, including cancer, cardiovascular disease, diabetes, HIV, derma-pathologies, urological illnesses, surgical interventions, mental disorders, and pulmonary diseases (26–30). It should be emphasized that, in addition to the advantages it offers, social media use also carries ethical and legal dangers, including the potential to violate patient confidentiality, harm healthcare professionals' reputations, and handle incomplete or inaccurate information. In addition, during the recent pandemic, social media was very effective in quickly disseminating knowledge among healthcare workers (31) and also for health promotion (32). The use of social media for improving knowledge and awareness of various healthcare workers including physicians, nurses, and other workers was investigated in various studies (23, 27, 33, 34); however, there were no studies identified in relation to the social media use by phlebotomists for knowledge exchange and increasing awareness. It is important to investigate this particular area so that it can help decision-makers in enabling the ethical, efficient, and effective use of social media in the workplace of phlebotomists for improving safety knowledge, which can result in improved patients' safety, improved quality of care, increased efficiency, better compliance with regulations, and also support the professional growth of phlebotomists. Therefore, this study aims to investigate the use of social media for the improvement of safety knowledge and awareness among phlebotomists.

## Methods

As this study was intended to arrive at specific conclusions using empirical evidence, a deductive quantitative cross-sectional design was adopted (35).

### Study setting and participants

This study explicitly focused on phlebotomists; therefore, 14 public hospitals from the Eastern region of Saudi Arabia were included. The data were collected using online survey platforms.

### Questionnaire design

The survey questionnaire in this study was partly developed by the authors. The questionnaire contains two sections. The first section focuses on collecting demographic information. The second section has 13 questions focusing on the use of social media. Among the 13 questions, 6 questions (7–12) were adopted from a similar study (36), while the rest were developed by authors. The survey questionnaire was designed in the English language as it can be easily understood by both Saudi and non-Saudi phlebotomists. The quality of the questions was reviewed by a panel of five academic professors from the health information management department at Imam Abdulrahman Bin Faisal University, Saudi Arabia. Furthermore, a pilot study was conducted with six phlebotomists at the university hospital. The results were analyzed, and the Cronbach alpha was identified to be >0.85 for all items, indicating good reliability and internal consistency (37). The survey questionnaire was then uploaded online using Google Forms, and the survey link was generated.

### Recruitment and sampling

After getting ethics approval from the university and permission from the hospital administration, the survey link was emailed to phlebotomists at the respective hospitals and also through social media applications. As the authors have accessed these hospitals considering the mobility and accessibility, focusing specifically on the eastern region, the approach reflects the adoption of convenience and purposive sampling techniques (38) in this study. The total number of phlebotomists at the selected hospitals (*n* = 724) was used in Cochran's formula to calculate the ideal sample required for the study (39), and it was calculated to be 252 at a 95% confidence interval.

### Data collection

As stated earlier, the survey link was forwarded to 724 phlebotomists. At the end of 4 weeks after forwarding the link, a total of 521 responses were collected. However, 86 responses were not fully completed. After the removal of incomplete responses, a total of 435 responses were considered for the data analysis.

### Ethics

Before commencing the survey, informed consent was obtained from each participant after they had been fully apprised of the study's objectives. The anonymity of the participants and their rights to the data were protected. All the ethical procedures prescribed at Imam Abdulrahman Bin Faisal University were followed. Ethics approval for the study was obtained from the Research Ethics Committee at the university.

### Data analysis

To analyze the overall data, statistical techniques including means, relative frequencies, and standard deviations were used as the majority of the data were numerical. Additionally, one-way ANOVA and *t*-tests were performed using Microsoft SPSS to compare the significant statistical differences between the participant groups.

## Results

As shown in Table 1, the participants were appropriately distributed across both sexes, including 49.2% male and 50.8% female phlebotomists. Considering the age group, 37.2% of the total participants belonged to the 30–39 years age group; 37% belonged to the 20–29 years age group; 20.2% to the 40–49 years age group; and 5.5% were aged 50 or more than 50 years. Focusing on education, the majority of the participants had a diploma degree (36.5%), followed by a bachelor's degree (26.4%), a master's degree (17.9%), other degrees (13.5%), and Ph.D. holders (5.5%). Given the work experience, the majority of the participants had more than 6 years of work experience, with 36.3% having more than 9 years of experience and 26.6% having 6 to 9 years of work experience. Approximately 27.8% had work experience of < 3 years, and only 9.1% had 3 to 6 years of work experience.

**Table 1:** Participant demographics.

	**Variables**	***N***
Sex	Male	214
	Female	221
Age group	20–29 years	161
	30–39 years	162
	40–49 years	88
	50–59 years	22
	>59 years	2
Education	Diploma	159
	Bachelor's degree	115
	Master's degree	78
	Ph.D.	24
	Others	59
Work experience	< 3 years	121
	3–6 years	40
	6–9 years	116
	>9 years	158

**Figure 1:**
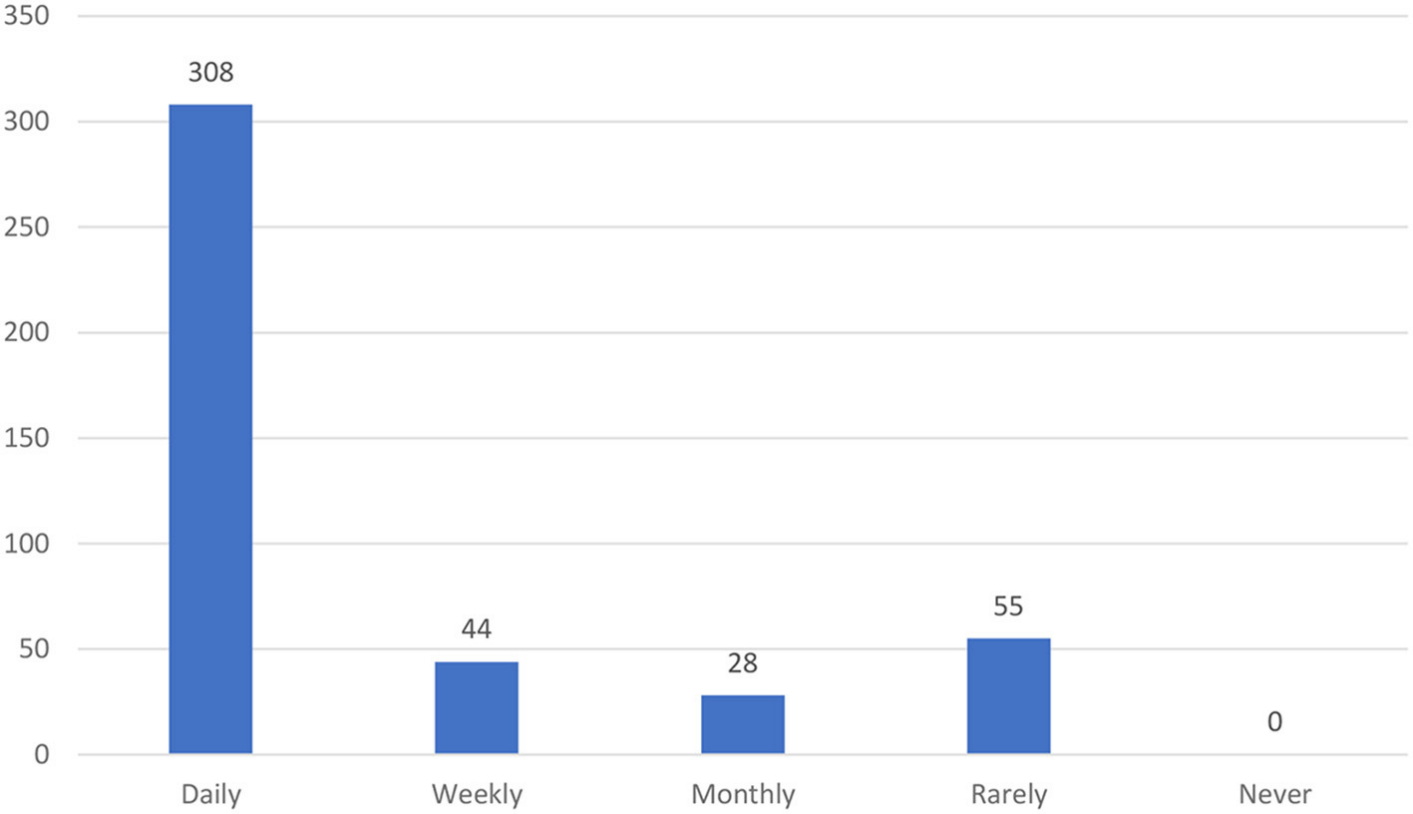
Frequency of social media usage.

**Figure 2:**
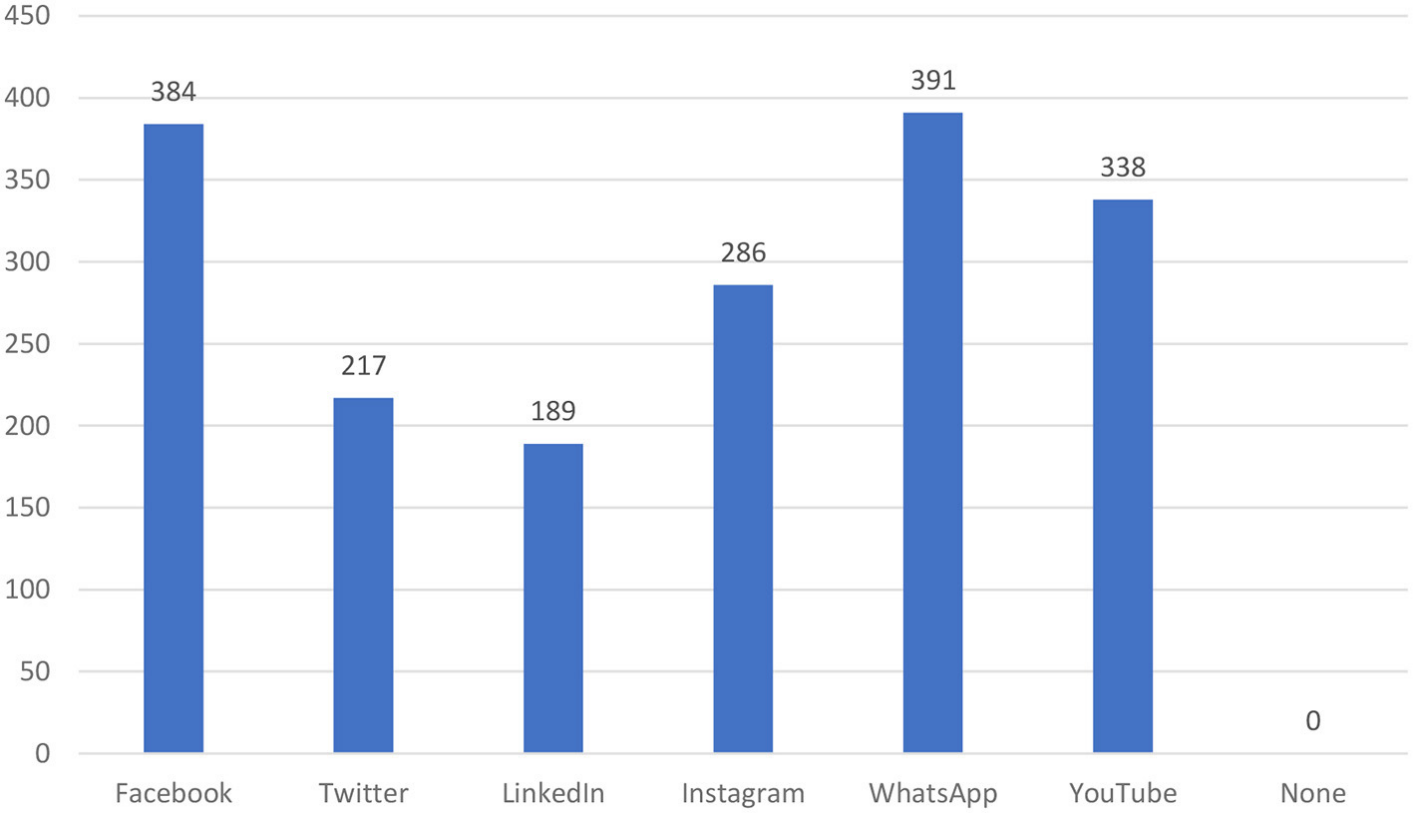
Social media applications used by the participants.

Analyzing the frequency of social media usage, it was identified that 70.8% of participants used daily, 10.1% used weekly, 6.4% used monthly, and 12.6% used rarely (see Figure 1). Among the social media applications used, most of them used more than two applications (see Figure 2). It is observed that WhatsApp was used by 89.9%; Facebook was used by 88.2%; YouTube was used by 89.1%; Instagram was used by 65.7%; Twitter was used by 49.8%; and LinkedIn was used by 43.4%.

**Figure 3:**
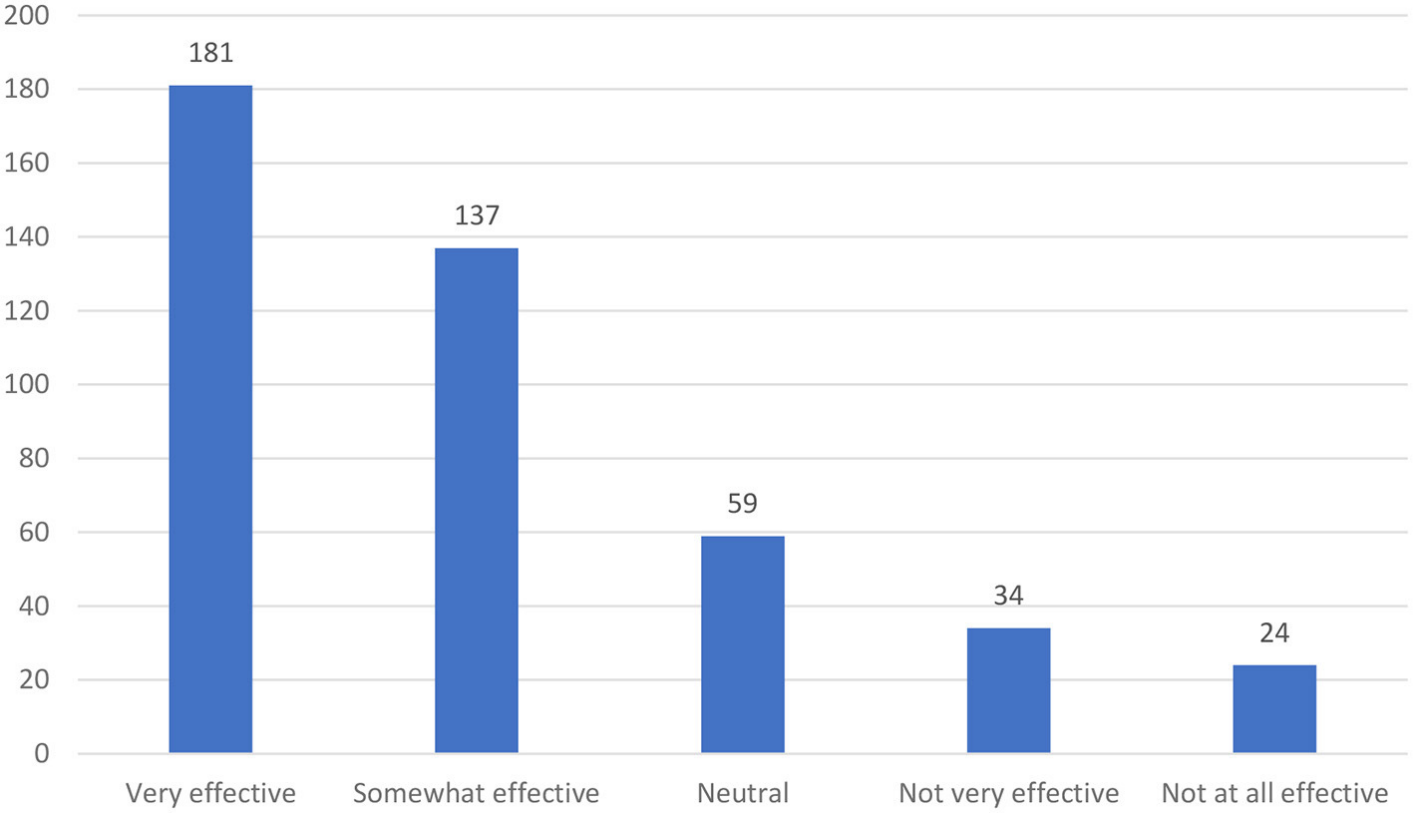
The effectiveness of social media applications in improving safety knowledge and awareness.

Approximately 60% of the total participants stated that they use social media applications for more than 3 h a day, while 28% use them for 1 to 2 h a day. In addition, 84.9% stated that they use social media for entertainment, 72.4% stated that they use social media for education, and 81.2% stated that they use social media for other purposes. Considering the effectiveness of social media in improving safety knowledge and awareness, 41.6% stated that it is very effective, and 31.5% stated that it was somewhat effective. However, only 14.7% stated that it is not very effective and not at all effective (see Figure 3).

**Figure 4:**
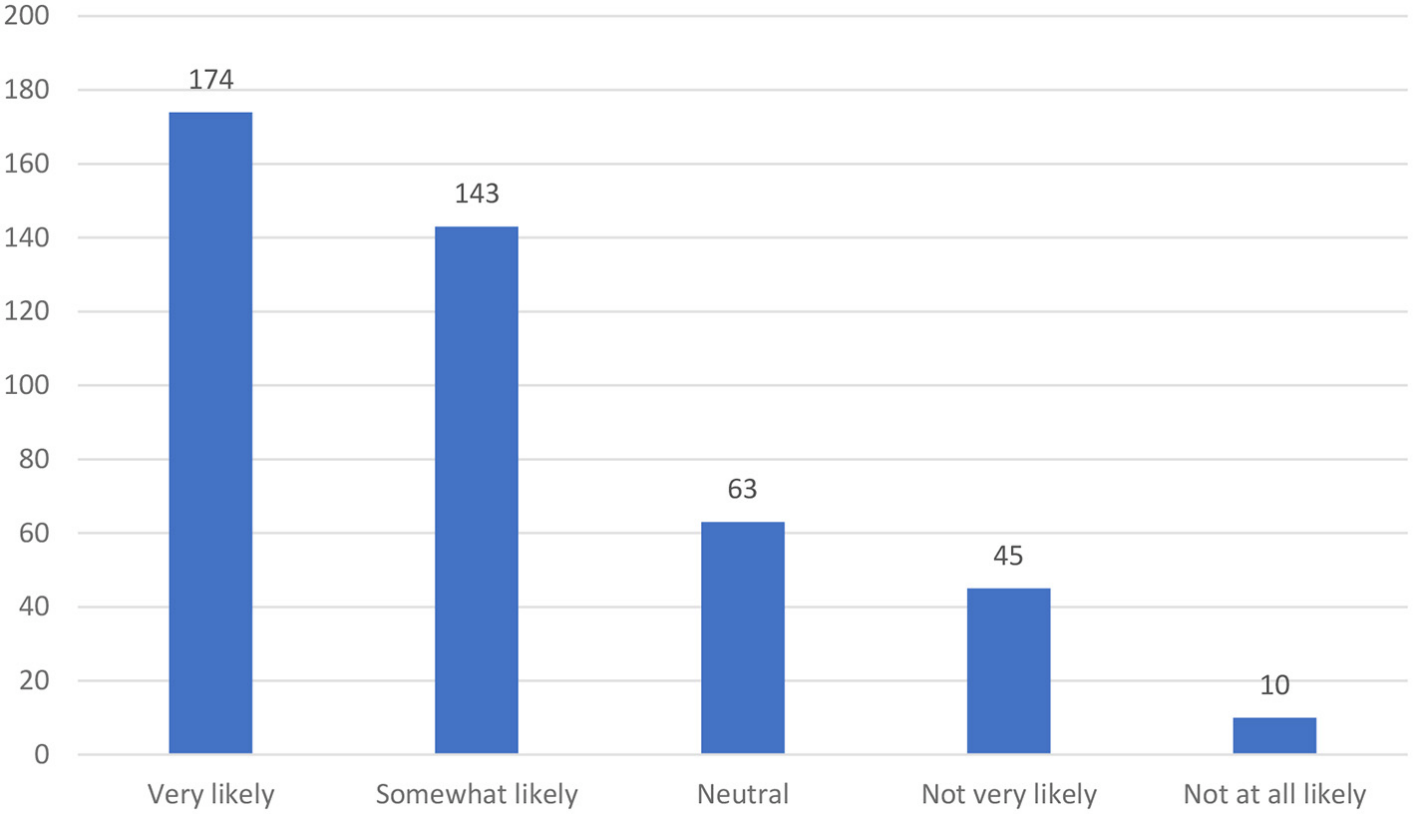
Likelihood of referring social media applications to colleagues for improving safety knowledge and awareness.

It is identified that 79.5% of the total participants stated that they learned new information about phlebotomy using social media applications. In addition, 76.3% of the participants stated that they share some information about phlebotomy with their colleagues using social media applications. Moreover, 72.8% of the participants (combined numbers of very likely and somewhat likely) stated that they are likely to refer social media applications to their colleagues for improving safety knowledge and awareness (see Figure 4).

**Figure 5:**
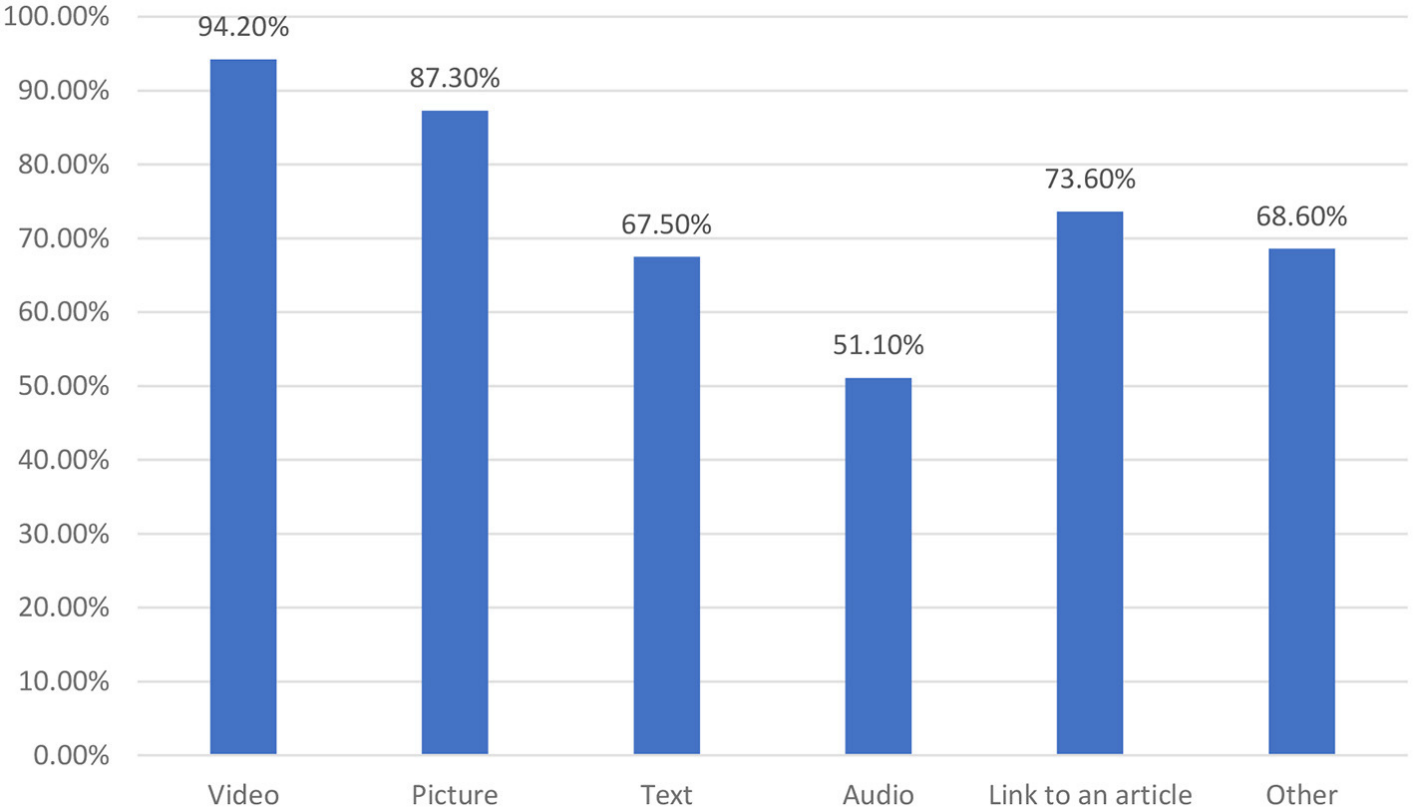
Preference of media formats for safety-related information.

For the preferences on media formats of accessing and viewing information, video and picture formats were preferred by more than 85% of participants, while only 51.1% preferred audio formats. Text formats and articles/journals were also preferred by more than 65% of the participants (see Figure 5).

Approximately 89.4% of the total participants stated that they would use social media if they need any information during working hours; in relation to the sources they preferred, 91.2% preferred the Internet and 88.7% preferred social media, followed by books (52.5%), journals (43.1%), short notes (26.2%), and others (19.8%).

Approximately 65.7% of the total participants stated that they are willing to increase social media use in professional practice about learning phlebotomy safety procedures and practices, while 12.6% stated that they may increase the usage in the future.

**Figure 6:**
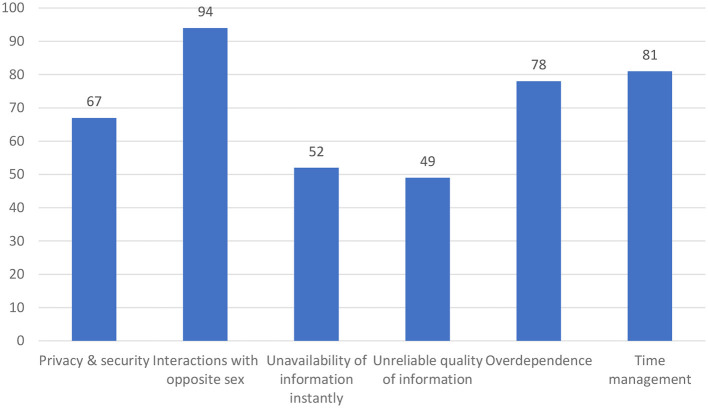
Challenges of using social media for sharing safety knowledge.

In addition, in relation to the drawbacks or challenges that the participants have experienced when using social media applications for learning about phlebotomy safety, some of the major factors identified (grouped similar opinions under a theme) are presented in Figure 6. Interactions with opposite sexes and time management were the most related comments observed.

Furthermore, to identify whether differences existed among the participants' groups based on sex, *t*-tests were conducted, and the results as shown in Table 2 revealed no major differences between the male and female participants with respect to the effectiveness of social media for improving safety knowledge and the intention to use them in the future.

**Table 2:** Differences between the sexes on the effectiveness of social media and intention to use it in the future.

**Factor**	**Groups**	***N***	**Mean**	**Std. Dev**.	***df***	***t-value***	***p-value***
Effectiveness	Male	214	3.92	1.64	410	0.5847	0.2759
	Female	221	3.99	1.09			
Intention to use it in the future	Male	214	2.52	0.65	428	1.5269	0.0637
	Female	221	2.64	0.56			

Similarly, Table 3 presents one-way ANOVA results to identify the differences among the age groups in relation to the effectiveness and intention to use in the future (see Table 3). Significant differences among the age groups were identified in relation to the effectiveness of social media (*p*<*0.05*) and intention to use it in the future (*p*<*0.05)*.

**Table 3:** Differences between the age groups on the effectiveness of social media and intention to use it in the future.

		**20–29 years**	**30–39 years**	**40–49 years**	**≥50 years**
Effectiveness	N	161	162	88	24
	∑X	760	602	336	24
	Mean	4.7205	3.716	3.8182	1
	∑X^2^	3620	2350	1414	24
	Std.Dev.	0.4502	0.8375	1.2275	0
	*F = 163.59753*				
	**p-value** **<** 0.00001^*^				
Intention to use it in the future	N	161	162	88	24
	∑X	456	467	173	29
	Mean	2.8323	2.8827	1.9659	1.2083
	∑X2	1336	1379	421	39
	Std.Dev.	0.5272	0.4512	0.9643	0.4149
	*F* = 91.65748				
	**p-value** **<** 0.00001^*^				

^*^Statistically significant.

## Discussion

This study has achieved its objective of analyzing the use of social media for the improvement of safety knowledge and awareness among phlebotomists. It is evident from the results that the majority of the participants used social media daily, and nearly 60% were using it for more than 3 h a day. Similar results were reported globally on the use of social media (1–5). These results indicate that phlebotomists use social media in the workplace more frequently, indicating that most phlebotomists are active social media users, and this identity can be used for enhancing safety knowledge and information-sharing behavior among themselves. Focusing on the social media platforms, WhatsApp, Facebook, and YouTube were the most used channels by the majority of the participants. As per 2022 statistics, in Saudi Arabia, 82.3% of the population uses social media, the country has 11.4 million Facebook users, and its ad can reach 40.7% of the eligible population, 29.3 million YouTube users with a reach of 84.1% population, and 15.45 million Instagram users in Saudi Arabia with a reach of 55.2% population (40), supporting the results identified in this study. Furthermore, a similar study conducted on radiologists in Saudi Arabia (36) has identified that WhatsApp was the most used social media application followed by YouTube, Snapchat, Twitter, Instagram, and Facebook. Focusing on the main factor, i.e., the effectiveness of social media for improving safety knowledge, more than 70% stated that it is effective, indicating the positive perceptions of phlebotomists in relation to social media for knowledge enhancement and information sharing. Recent studies (41–44) have suggested that social media can be effective in improving knowledge, information sharing, and communication among various healthcare workers, similar to the findings achieved for phlebotomists in this study. The effectiveness can also be understood from the high extent of learning new information by the participants using social media, with more than 70% agreeing that they frequently share information about phlebotomy or relevant updates.

As observed from the results, the preference for information formats mostly reflected videos, pictures, and journals/articles. Social media applications can support all types of information formats, and similar results were identified in (36). During work hours, it was evident that 89% of participants were using social media for accessing new information, but when asked about sources, 91.2% preferred the Internet (browser application or other applications) and 88.7% preferred social media. Therefore, it is clear that other applications are used in the workplace apart from social media. Considering this factor, it is essential that phlebotomists are made aware of the effectiveness of social media for knowledge sharing. Moreover, it is interesting to observe that more than 65% of participants were currently willing to increase their social media usage. This has to be considered as an opportunity to integrate social media channels into healthcare for increasing safety knowledge among healthcare workers. One of the interesting findings is the challenges associated with opposite-sex interaction on social media, followed by time management. Although the issue seems to be a normal issue, in a conservative country such as Saudi Arabia, the laws are strict, and family norms do not encourage opposite-sex interaction with unknowns (45, 46), which could create hurdles in information exchange and communication via social media among phlebotomists.

Furthermore, the results of the *t*-test did not identify any difference between the male and female participants, indicating that both sexes perceived the high effectiveness of social media for improving safety knowledge and awareness, and also in their intention to use social media for information in the future. However, in relation to effectiveness, younger participants stated that social media is more highly effective compared to older participants. Similarly, younger participants were more interested in using social media in the future, suggesting that young phlebotomists more actively support and use social media compared to older phlebotomists. Therefore, there is a need to provide training for older phlebotomists to create awareness about the benefits of social media for improving knowledge. Overall, the findings have indicated the positive effect of the use of social media on improving safety knowledge among phlebotomists.

Although the results revealed the effectiveness of social media in contributing to the knowledge and awareness of phlebotomists, it is important to consider the issue of health misinformation on social media platforms, which can lead to equally negative outcomes. It was observed that people with all personality traits shared misinformation about COVID-19 during the pandemic. However, it was observed that users with high cognitive levels shared misinformation to a lesser extent than users with low cognitive levels (47, 48). It is also possible that healthcare professionals may access health misinformation on social media and spread it unintentionally if they lack awareness and analytical skills. A recent study (48) observed that healthcare professionals were similar to healthcare students in correctly distinguishing between true and false news stories. However, skills including analytical and open-minded thinking were positively correlated with the ability to distinguish true and false information. In addition, healthcare professionals have a tendency to believe that news articles are true if they are consistent with the narratives that have already been established (49). Therefore, it is important for all the stakeholders including healthcare professionals, institutions, government, and the public to be vigilant and take responsibility in addressing the issue of misinformation by establishing transparency and trust through reliable communication channels, either through their own platforms or through personalized social media pages (50). The responsibility of healthcare professionals is largely recognized in a recent study as they can help patients and fellow citizens obtain reliable, evidence-based health information (51). Focusing on the government, the policies adopted may be complex to analyze. For instance, in Saudi Arabia, individuals who intentionally share misinformation are fined up to SAR 3 million (approximately US $800,000) and can be jailed for up to 5 years (52). While it is important to adopt strict laws and regulations, the essential focus should be on improving the skills, knowledge, and awareness of identifying misinformation among all stakeholders as there are high chances of arising legal complexities in proving the unintentional action in sharing the misinformation. Therefore, it is essential to adopt various strategies for managing misinformation, some of which are included below:

- Media Literacy Programs: Support media literacy initiatives that educate individuals about evaluating information credibility, identifying biases, and understanding the scientific process. Encourage schools, universities, and community organizations to integrate media literacy programs into their curriculum or public outreach efforts.
- Support from Regulatory Bodies: Advocate for the development and enforcement of regulations related to health misinformation on social media. Work with regulatory bodies and policymakers to create guidelines and policies that hold individuals and organizations accountable for spreading false health information.
- Continuous Monitoring and Adaptation: Regularly monitor social media platforms for emerging health misinformation trends and adapt strategies accordingly. Stay up to date with the latest research, developments, and best practices in combating health misinformation to ensure the effectiveness of one's efforts.
- Collaboration with Social Media Platforms: Collaborate with social media platforms to develop policies and mechanisms that address health misinformation effectively. Advocate for algorithms that prioritize accurate and reliable information while downplaying or flagging misleading content. Engage in dialogue with platform representatives to highlight the impact of health misinformation on public health.
- Educate the Public: Conduct awareness campaigns on the risks of health misinformation and the importance of relying on trusted sources. Educate the public about the potential consequences of acting on inaccurate health information. Promote critical thinking skills and empower individuals to become discerning consumers of health-related content.
- Reporting and Flagging: Encourage users to report and flag health misinformation on social media platforms. Many platforms have mechanisms for reporting false or misleading content. By reporting such content, users can contribute to the removal of misinformation and raise awareness among platform moderators.
- Fact-Checking: Promote the importance of fact-checking and critical thinking among social media users. Share resources and tools that help individuals verify the accuracy of health information. Encourage users to check the credibility of sources, look for supporting evidence, and consult reputable health organizations or professionals.
- Collaborate with Experts: Partner with healthcare professionals, medical organizations, and reputable sources to amplify credible voices. Collaborations can include hosting live Q&A sessions, interviews, or panel discussions with experts to address common health concerns and debunk misinformation. Their authoritative expertise adds credibility to the information being shared.

### Implications

Although no study was found to evaluate the use of social media to increase phlebotomists' knowledge, numerous studies have found the substantial usefulness of social media in spreading information to various other healthcare professionals in hospitals. Based on the aforementioned findings, the study's most important conclusion was that the majority of participants thought social media may assist in spreading knowledge to advance phlebotomists' skills, understanding, and awareness of safety. These findings can help policymakers create social media adoption strategies that will promote knowledge and information sharing at work, which will lead to better professional growth and healthcare quality. Additionally, the issues brought up—such as privacy and security, relationships between people of different sexes, inaccurate or low-quality information, and time management—give decision-makers an overview of the issues that can be solved through the development of policies. This study also adds to the body of knowledge by illuminating the advantages and difficulties of using social media for patient care and professional growth.

### Limitations

The significant drawback of this study was that the majority of participants came from a small sample of the eastern region of Saudi Arabia, indicating that more effort needs to be put in future research to include participants from all parts of the Kingdom of Saudi Arabia. It could be interesting to investigate any statistical relationships between demographic variables and the use of social media for knowledge dissemination. Additionally, it is advised to establish and assess the performance of a WhatsApp group and other popular applications in the region for the purpose of disseminating information on phlebology safety and raising awareness among phlebotomists in Saudi Arabia.

## Conclusion

The findings indicate that the majority of respondents thought social media may assist in spreading knowledge about safety among phlebotomists in Saudi Arabia. As a result, they are eager to expand the amount of time they spend on social media for this reason in their future professional endeavors. Furthermore, the assessment of the literature revealed that there have only been a few international studies on the use of social media to improve healthcare workers' awareness of safety knowledge and no studies associated with phlebotomists.

## Data availability statement

The raw data supporting the conclusions of this article will be made available by the authors, without undue reservation.

## Ethics statement

Before commencing the survey, an informed consent was obtained from each participant after they had been fully apprised of the study's objectives. Ethical approval for the study was obtained from the Research Ethics Committee at the Imam Abdulrahman Bin Faisal university.

## Author contributions

All authors listed have made a substantial, direct, and intellectual contribution to the work and approved it for publication.
